# Increased *MET* gene copy number negatively affects the survival of esophageal squamous cell carcinoma patients

**DOI:** 10.1186/s12885-019-5450-6

**Published:** 2019-03-18

**Authors:** Yanqiu Wang, Zhengzeng Jiang, Chen Xu, Hao Wang, Lijie Tan, Jieakesu Su, Xin Wang, Dongxian Jiang, Yingyong Hou, Qi Song

**Affiliations:** 1 Department of Pathology, Zhongshan Hospital, Fudan University, Shanghai, 200032 People’s Republic of China; 2 Department of Thoracic surgery, Zhongshan Hospital, Fudan University, Shanghai, 200032 People’s Republic of China; 3 Department of Pathology, School of Basic Medical Sciences & Zhongshan Hospital, Fudan University, Shanghai, 200032 People’s Republic of China; 4 Department of Pathology, Qingpu Branch of Zhongshan Hospital, Fudan University, Shanghai, 201700 People’s Republic of China

**Keywords:** Increased *MET* gene copy number, Esophageal squamous cell carcinoma (ESCC), Prognosis, Clinical stage, Fluorescence in situ hybridization (FISH)

## Abstract

**Backgrounds:**

Since *Mesenchymal epithelial transition* (*MET*) amplification has been regarded as a potential treatment target, the knowledge of its prevalence and prognostic importance is crucial. However, its clinical pathologic characteristics are not well known in esophageal squamous cell carcinoma (ESCC).

**Methods:**

We investigated *MET* gene status with fluorescence in situ hybridization (FISH) assay in 495 ESCC cases using tissue microarrays. Prognostic significance as well as correlations with various clinicopathological parameters was evaluated.

**Results:**

Among 495 patients, 28 (5.7%) cases were *MET* FISH positive, including 5 cases (1%) with true gene amplification. There were no statistically significant associations between *MET* FISH-positivity and clinicopathologic characteristics. A significantly poorer prognosis was observed in 28 patients with *MET* FISH-positivity (disease free survival/DFS, *P* < 0.001 and overall survival/OS, *P* = 0.001). Multivariate analysis revealed *MET* FISH-positivity was an independent prognostic factor for DFS (hazard ratio/HR, 1.953; 95% confidence interval/CI, 1.271–2.999; *P* = 0.002) and OS (HR, 1.926; 95% CI, 1.243–2.983; *P* = 0.003). *MET* FISH-positivity was associated with DFS (*P* = 0.022 and 0.020) and OS (*P* = 0.046 and 0.024) both in stage I-II ESCC and in stage III-IVa ESCC. No statistical significance (DFS, *P* = 0.492 and OS, *P* = 0.344) was detected between stage I-II ESCC with *MET* FISH-positivity and stage III-IVa ESCC with FISH-negativity.

**Conclusions:**

Increased *MET* gene copy number is an independent prognostic factor in ESCC, and ESCC might have potentially been up-staged by increased *MET* gene copy number. The results indicate that increased *MET* gene copy number is a very promising parameter, in clinical therapy and follow-up plans.

## Background

Esophageal cancer (EC) is the ninth most common cancer and the sixth leading causes of cancer death globally [1]. In China, there were about 477,900 newly diagnosed EC (the third most commonly cancers), and about 375,000 cases dead of EC (the fourth leading causes of cancer death) in 2015 [2]. Esophageal squamous cell carcinoma (ESCC) is the most common histological subtype of EC. In China, approximately 90% of EC are ESCC [3]. Despite the improvement in the traditionally therapeutic management for ESCC, the prognosis of some patients remains dismal [4]. Therefore, the identification of prognostic factors in these patients may be of great importance. Despite Tumor-node-metastasis (TNM) stage is the most important conventional prognostic factor in tumors, evidence is increasing that patients’ prognosis depends not only on tumor stage, but also on the tumor-specific molecular alteration [1]. Recent advancements in molecular biology have made it possible to detect molecular alteration in human tumors, and molecular prognostic markers are subjects of intense research [5–7].

*Mesenchymal epithelial transition* (*MET*) gene was first identified in 1984 in an osteosarcoma immortalized cell line [8]. As a proto-oncogene located on chromosome 7q31.2, it encodes a heterodimeric transmembrane receptor with tyrosine kinase activity (RTK) for the hepatocyte growth factor (HGF). MET activation triggers a variety of downstream signaling pathways, such as the PI3K/AKT/mTOR and RAS/ERK/MAPK pathways [9]. Normal MET activation is required for embryogenesis, cell growth, cell differentiation and angiogenesis. Aberrant MET activation has been reported in various types of cancer, and promotes tumor cell proliferation, motility, invasion and metastasis. The abnormally activating mechanism typically involves *MET* gene amplification, Met and/or HGF protein overexpression, or, rarely, domain-specific sequence mutations [10, 11].

Recent studies found different tumors with *MET* amplification were extraordinarily susceptible to the selective MET tyrosine kinase inhibitor (TKI) [12–14], and *MET* amplification was responsible for approximately 20% of the acquired resistance to epidermal growth factor receptor (EGFR) TKI treatment in lung adenocarcinomas [15, 16]. The inspiring findings trigger investigators to explore the prevalence and clinical relevance of *MET* gene amplification in different tumors. *MET* gene amplification is identified in 2–5% of gastric cancers [17, 18], 2–4% of esophageal adenocarcinoma (EAC) [5, 12], 1–8% of non-small cell lung cancer (NSCLC) [10, 13, 19], and 2–10% of colorectal cancers [13, 20]. And *MET* amplification is thought to be associated with metastasis and poorer outcome in gastric [21], lung [22] and colorectal cancers [23]. Despite the great interest on *MET* amplification, only few small studies evaluated its gene status in ESCC [24].

Therefore, in this study, we aimed to evaluate *MET* gene copy status in a large cohort of ESCC. In addition, we sought to analyze its clinicopathological features and prognostic value.

## Methods

### Patients

This retrospective study was conducted in a cohort of 495 treatment-naive ESCC patients who underwent esophagectomy at Zhongshan Hospital between January 2007 and December 2010. Patients were included in the study if the following criterias were met: (1) underwent primary resection, (2) with no prior treatment, and (3) with available complete medical records. Patients were excluded from the study if they had disease progression within three months after surgery. Clinical and histopathological data, including sex, age, smoking status, tumor size, tumor location, differentiation, vessel or nerve invasion, pT stage, and pN stage, was obtained from the patients’ medical and pathological records. The pathologic tumor-node-metastasis (pTNM) stage was performed according to the 8th edition of the American Joint Committee on Cancer (AJCC) staging system. All patients were followed up every 3–6 months after tumor resection, and patients underwent follow-up examinations to identify possible tumor recurrence. Exam methods included endoscopy, computed tomography, magnetic resonance imaging, abdominal ultrasonography, and measurement of serum tumor marker levels.

Written informed consent was obtained from all patients, and the study was approved by the ethical committee of the Zhongshan Hospital, in accordance with the ethical standards of the World Medical Association Declaration of Helsinki.

### Tissue microarrays (TMAs)

TMA construction was performed as previously described [25]. Briefly, histological sections were examined by a pathologist, and representative tumor areas free from necrosis or hemorrhage were pre-marked in formalin-fixed paraffin-embedded (FFPE) donor blocks. Two or three core tissues (2 mm in width and 6 mm in length) from different representative areas per case were taken from the donor blocks and arranged in recipient blocks (tissue array blocks). Our TMAs contained the tumor samples, several normal esophagus and other control tissues.

### Fluorescence in situ hybridization (FISH)

*MET* gene status was evaluated using a commercially available FISH assay [26], with Vysis *MET* Spectrum Red FISH Probe (Abbott Molecular, Chicago, IL, USA) and control Vysis *CEP7* Centromere Spectrum Green Probe (Abbott Molecular) on 4 μm-thick TMA sections. The signals of each sample were counted in at least 50 well-defined nuclei using a fluorescence microscope (BX43, Olympus, Tokyo, Japan) equipped with a Microscope Digital Camera (DP73, Olympus, Tokyo, Japan). An average *MET* gene copy number ≥ 5 and a *MET/CEP7* ratio ≥ 2 (true *MET* amplification) were regarded as *MET* FISH positive [22].

### Statistical analysis

The Chi square and Fisher’s exact tests were used to evaluate the association between *MET* status and clinicopathological characteristics. The primary and secondary endpoints were cancer-related death and recurrence/metastasis. Disease free survival (DFS) and overall survival (OS) were defined as periods from the date of surgical treatment until the date of disease progression (event: recurrence, metastasis, deaths) and the date of cancer-specific survival (event: cancer-related death), respectively. The Kaplan–Meier analysis with the log-rank test was performed to determine the prognostic significance for DFS and OS. The univariate and multivariate Cox proportional hazard regression analysis was used to identify the independent prognostic factors. The hazard ratio (HR) and its 95% confidence interval (CI) were assessed for each factor.

Statistical analysis was carried out using SPSS 21.0 statistical software (SPSS, Chicago, IL, USA). All tests were two sided, and *P*-values < 0.05 were considered to be statistically significant.

## Results

### Clinical data

The patients’ clinicopathological characteristics are summarized in Table 1. The patient group consisted of 408 men (82.4%) and 87 women (17.6%) with a median age of 61 years (range, 34–83 years). One hundred ninety-nine subjects (40.2%) were ever-smokers or smokers, whereas 296 (59.8%) were nonsmokers. The mean tumor size was 3.4 cm. By anatomic site, 47.9% of tumors were located in the lower esophagus, 47.0% in the middle esophagus, and 5.1% in the upper esophagus. The tumors were poorly differentiated in 40.2%, moderately differentiated in 56.0%, and well differentiated in 3.8%. Vessel and nerve invasion were identified in 110 (22.2%) and 178 (36.0%) tumors, respectively. There were 9.3% patients at pathologic stage T1, with 22.2, 68.3, and 0.2% at stages T2, T3, and T4, respectively. About pathologic N stages, there were 53.3, 25.9, 15.8, 5.1% patients at N0, N1, N2, and N3 stages respectively. According to the 8th edition of TNM staging, 38 patients (7.7%) were classified as having stage I disease, 234 patients (47.3%) as stage II, 193 patients (39.0%) as stage III, and 30 patients (6.1%) as stage IVa.

**Table 1:** Correlation between *MET* FISH-positivity and ESCC clinicopathological parameters

	MET FISH-positivity			
	Number	No	Yes	*P* value
Sex				0.638
Female	87	83	4	
Male	408	384	24	
Age				0.932
< 60	216	204	12	
≥60	279	263	16	
Smoking				0.919
No	296	279	17	
Yes	199	188	11	
Tumor Size				0.434
< 3.4	283	265	18	
≥3.4	212	202	10	
Tumor Location				0.941
Upper	24	23	1	
Middle	220	207	13	
Lower	224	211	13	
Differentiation				0.957
Well	19	18	1	
Middle	277	262	15	
Poor	199	187	12	
Vessel invasion				0.194
No	385	366	19	
Yes	110	101	9	
Nerve invasion				0.706
No	317	300	17	
Yes	178	167	11	
pT				0.883
T1	46	44	2	
T2	110	105	5	
T3	338	317	21	
T4	1	1	0	
pN				0.088
N0	264	252	12	
N1	128	119	9	
N2	78	75	3	
N3	25	21	4	
Clinical stage				0.351
I-II	272	259	13	
III-IVa	223	208	15	
Disease progression				0.002
No	226	221	5	
Yes	269	246	23	
Cancer-related death				0.005
No	234	228	6	
Yes	261	239	22	

### Increased MET gene copy number

**Fig. 1:**
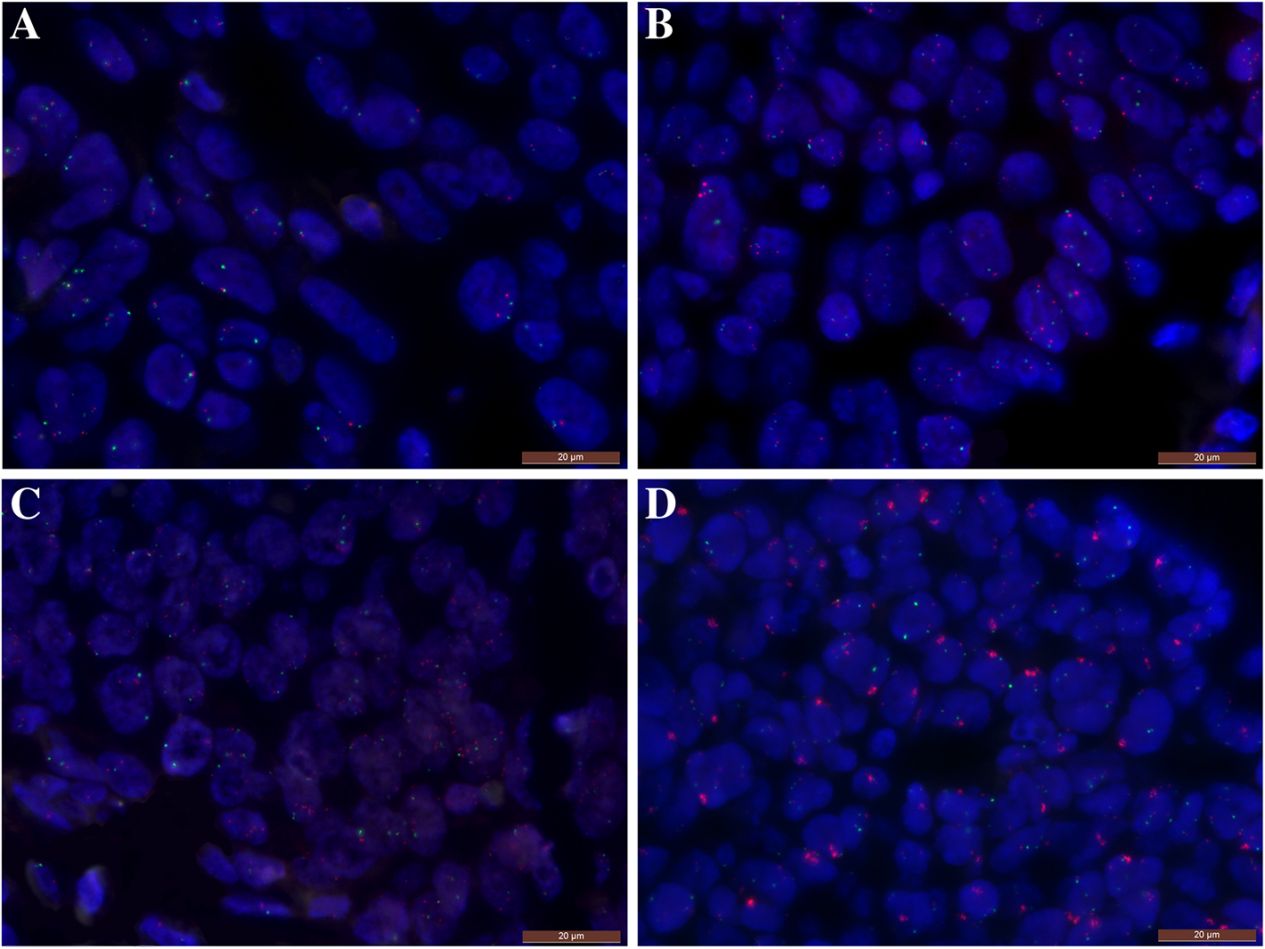
Representative microscopic images of *MET* (red) and CEP7 (green) fluorescence in situ hybridization. A, Normal gene status; B, *MET* low polysomy; C, *MET* FISH-positivity (an average number of *MET* signal per nucleus ≥5.0) and D, *MET* gene amplification (MET: CEP7 ratio of ≥2.0)

Among 495 patients, 28 (5.7%) cases were *MET* FISH positive (an average number of *MET* signals per nucleus ≥5.0), including 5 cases (1%) with true gene amplification (5 cases with *MET: CEP7* ratio of ≥2.0) (Fig. 1c and d). Other specimens showed disomy or low polysomy (94.3%) (Fig. 1a and b).

The correlations between *MET* FISH-positivity and clinical pathologic characteristics are listed in Table 1. *MET* FISH-positivity was significantly associated with DFS (2.2% in patients without disease progression vs. 8.6% in patients with disease progression, *P* = 0.002) and OS (2.6% vs. 8.4%, *P* = 0.005). However, there were no statistically significant difference in sex (*P* = 0.638), age (*P* = 0.932), smoking (*P* = 0.919), tumor size (*P* = 0.434), tumor location (*P* = 0.941), differentiation (*P* = 0.957), vessel invasion (*P* = 0.194) and nerve invasion (*P* = 0.706), pT stage (*P* = 0.883), pN stage (*P* = 0.088), and clinical stage (*P* = 0.351).

### Survival analysis

The median follow-up time was 35.0 months (range 3–102 months). Two hundred sixty-nine patients (54.3%) had disease progression and two hundred sixty-one patients (52.7%) had died from esophageal cancer during the follow-up. The 5-year DFS and disease-specific OS rates for all patients were 44.1 and 44.4%, respectively.

**Fig. 2:**
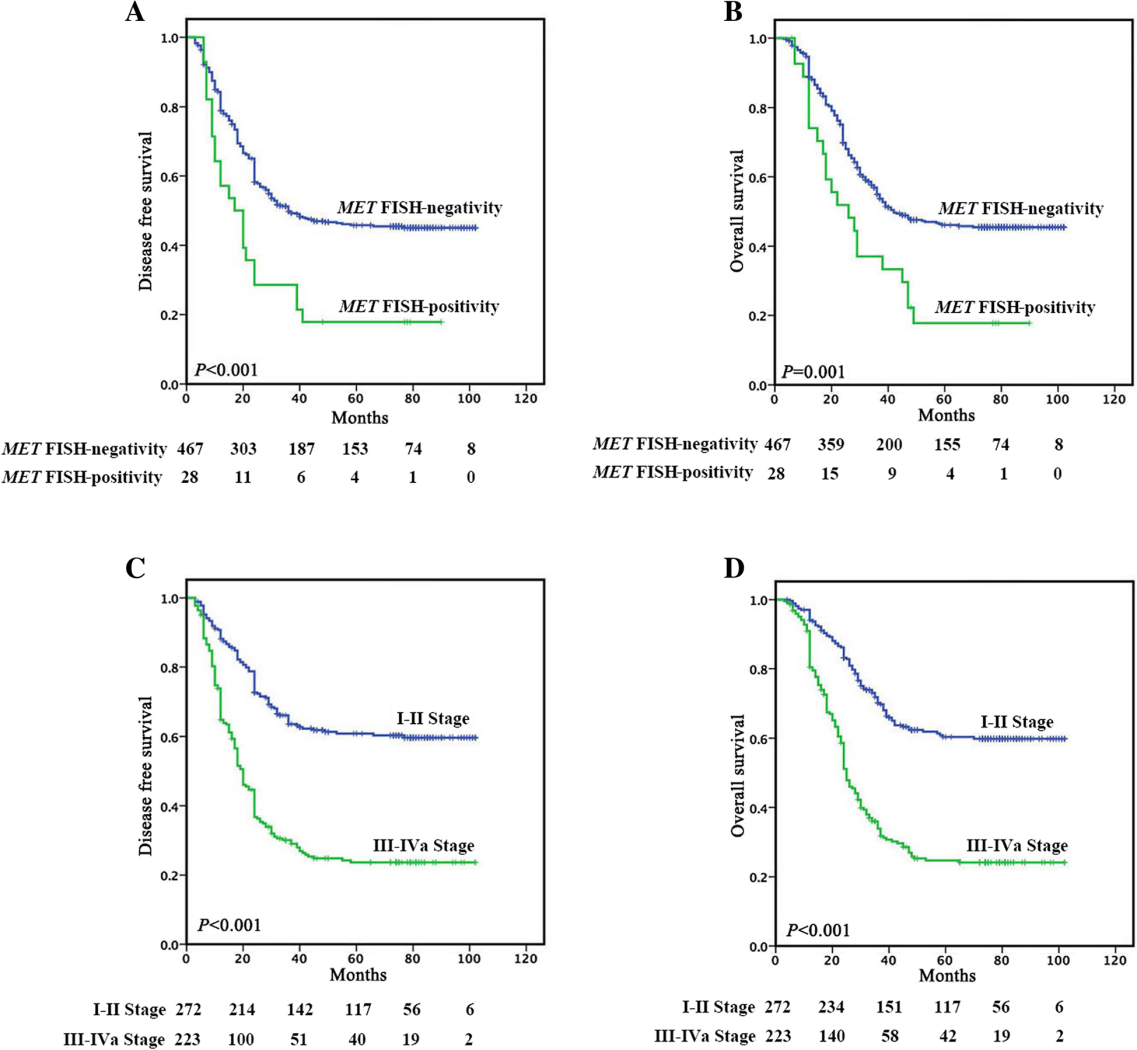
Kaplan-Meier Survival Analysis for DFS and OS in ESCC according to (A, B) *MET* FISH-positivity and (C, D) clinical stage, respectively

Figure 2a and b reveals that a significantly poorer prognosis was observed in 28 patients with *MET* FISH-positivity, showing a median DFS or OS of 17.0 or 26.0 months, respectively, compared with 36.0 or 42.0 months in the group with *MET* FISH-negativity (*P* < 0.001 or *P* = 0.001). The 5-year DFS (17.9%) and OS (17.8%) rates for patients with *MET* FISH-positivity were significantly lower than the corresponding rates (45.7 and 46.0%) for patients with *MET* FISH-negativity. Univariate analysis indicated that *MET* FISH-positivity, differentiation, vessel invasion, nerve invasion and clinical stage had significant impacts on DFS, and *MET* FISH positive, vessel invasion, nerve invasion and clinical stage had significant impacts on OS (both *P* < 0.05). Multivariate analysis revealed *MET* FISH-positivity was an independent prognostic factor for DFS (HR, 1.953; 95% CI, 1.271–2.999; *P* = 0.002) and OS (HR, 1.926; 95% CI, 1.243–2.983; *P* = 0.003). Clinical stage was also found to be an independent prognostic factor for DFS and OS (Table 2).

**Table 2:** Univariate and Multivariate Analysis for DFS and OS in ESCC Patients

	DFS		OS	
	*P* value	HR (95% CI)	*P* value	HR(95% CI)
Univariate analysis				
Sex	0.251	1.204 (0.877–1.655)	0.125	1.295 (0.931–1.802)
Age	0.994	1.001 (0.787–1.273)	0.982	0.997 (0.781–1.273)
Smoking	0.538	1.079 (0.846–1.377)	0.345	1.126 (0.880–1.440)
Tumor Size	0.166	1.185 (0.932–1.509)	0.113	1.218 (0.954–1.555)
Tumor Location	0.879	0.984 (0.799–1.212)	0.793	1.029 (0.831–1.274)
Differentiation	0.047	1.246 (1.003–1.549)	0.080	1.217 (0.977–1.518)
Vessel invasion	< 0.001	1.597 (1.228–2.076)	0.001	1.576 (1.205 2.061)
Nerve invasion	0.02	1.335 (1.046–1.703)	0.008	1.401 (1.094–1.793)
Clinical stage	< 0.001	2.856 (2.230–3.659)	< 0.001	2.899 (2.255–3.727)
*MET* FISH-positivity	0.001	2.114 (1.378–3.245)	0.002	2.002 (1.293–3.099)
Mutivariate analysis				
Differentiation	0.376	1.106 (0.885–1.381)	–	–
Vessel invasion	0.425	1.119 (0.849–1.474)	0.455	1.113 (0.841–1.472)
Nerve invasion	0.506	1.089 (0.848–1.398)	0.269	1.153 (0.896–1.485)
Clinical stage	< 0.001	2.672 (2.061–3.465)	< 0.001	2.745 (2.111–3.569)
*MET* FISH-positivity	0.002	1.953 (1.271–2.999)	0.003	1.926 (1.243–2.983)

### Survival analyses based on clinical stage

In stage I-II patients, one hundred four patients (38.2%) had disease progression and one hundred one patients (37.1%) had died from esophageal cancer during the follow-up. In stage III-IVa patients, one hundred sixty-five patients (74.0%) had disease progression and one hundred sixty patients (71.7%) had died from esophageal cancer during the follow-up.

Figure 2c and d reveals that a significantly poorer prognosis was observed in 223 stage III-IVa patients, showing a median DFS of 20.0 months or OS of 25.0 months, respectively, compared with not-reached median survival in 272 stage I-II patients (*P* < 0.001). The 5-year DFS (23.7%) and OS (24.7%) rates for stage III-IVa patients, were significantly lower than the corresponding rates (60.8 and 60.4%) for stage I-II patients.

**Fig. 3:**
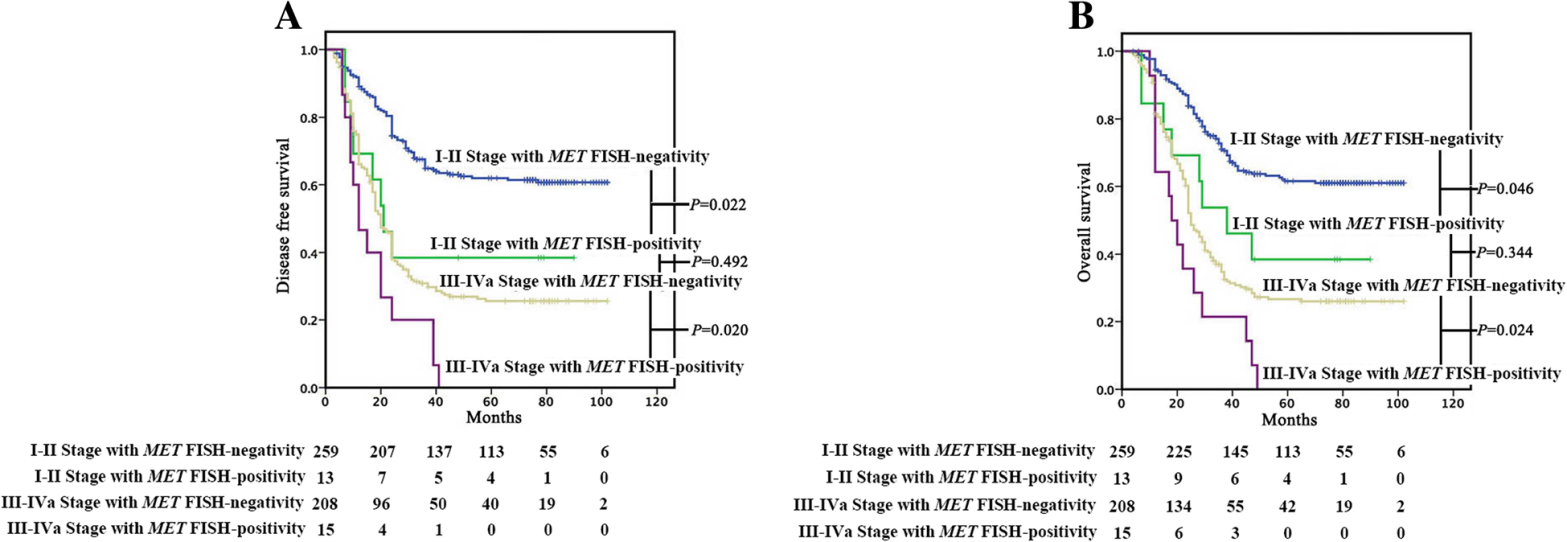
Kaplan-Meier Survival Analysis for DFS (A) and OS (B) in ESCC based on clinical stage and *MET* FISH-positivity

*MET* FISH-positivity was associated with DFS (*P* = 0.022) and OS (*P* = 0.046) in patients with stage I-II ESCC (Fig. 3a and b). In detail, a poorer prognosis was observed in 13 patients with *MET* FISH-positivity, with a median DFS or OS of 21.0 or 38.0 months, respectively, while those with *MET* FISH-negativity (*n* = 259) did not reach the median survival. *MET* FISH-positivity was also associated with DFS (*P* = 0.020) and OS (*P* = 0.024) in patients with stage III-IVa ESCC (*n* = 223) (Fig. 3a and b). In detail, a poorer prognosis was observed in 15 patients with *MET* FISH-positivity, with a median DFS or OS of 12.0 or 18.0 months, respectively, while those with *MET* FISH-negativity (*n* = 208), with a median DFS or OS of 20.0 or 25.0 months, respectively. What’s more, no statistical significance (DFS, *P* = 0.492 and OS, *P* = 0.344) was detected between stage I-II ESCC with *MET* FISH-positivity and stage III-IVa ESCC with FISH-negativity.

## Discussion

In our study, *MET* gene status was detected in 495 ESCC patients by FISH method. FISH analysis is a semiquantitative method that can be performed with two probes for determination of the number of signals for a target gene and for the centromere of the corresponding chromosome [27]. Comparing with southern blot and PCR-based methods, FISH has several advantages over other methods. It can be applied to FFPE tumor tissues for routine pathologic diagnosis, and is now widely used in clinical practice for the detection of gene amplification [28–30].

Our findings showed *MET* FISH positive rate was 5.7% and gene amplification rate was 1% using Cappuzzo criteria, which was consistent with the somatic copy number alteration data generated by The Cancer Genome Atlas Research Network [5]. As has been published previously in other tumors [31–33], the rate of *MET* amplification is relatively low. *MET* genetic alterations were detected using increasing gene copy number. The increasing gene copy number can result from mainly two genetic mechanisms [34]: 1) polysomy, a copy number gain, due to extra copies of the entire chromosome; and 2) gene amplification, the amplification of specific gene or a group of genes in a given chromosome. In 2009, Cappuzzo et al. found the survival outcome of patients with a mean *MET* gene copy number per cell higher than 5 and higher than 6 was similar, and worse than the other four groups with a mean copy number lower than 5 in NSCLC [22]. Gradually, the Cappuzzo criteria (*MET /CEP7* ratio ≥ 2.0 and/or *MET* ≥ 5.0 copies) has been widely accepted and used in other tumors, such as NSCLC [10, 35], gastric cancer [21, 36], gastroesophageal adenocarcinoma [17], tonsillar squamous cell carcinoma [37], and mesothelioma [38].

Since Lennerz etal has demonstrated that 2% of patients (10/489) with esophagogastric adenocarcinoma, who harbored *MET* amplification and were treated with a *MET* inhibitor, experienced tumor shrinkage in 2011 [12], *MET* gene status has gained considerable interest in solid tumors [13, 14]. Increased *MET* gene copy number has an established prognostic role in NSCLC, gastric cancer and gastroesophageal adenocarcinoma patients [17, 21, 39, 40]. However, its clinical pathologic characteristics are not well known in ESCC [24, 41], and to our knowledge, no previous study with a large number of ESCC has been reported. Our data demonstrated that 28 patients with *MET* FISH-positivity had a significantly worse DFS and OS than 467 individuals with FISH-negativity. Moreover, *MET* FISH-positivity was an independent prognostic factor for both DFS and OS, further indicating increased *MET* gene copy number is a negative prognostic factor in ESCC.

Subgroup analyses according to the disease stage were also conducted in our study. Lee et al. reported in gastric cancer, *MET* amplification did not have an impact on prognosis in early TNM stage (stage I or II), unlike in advanced TNM stage (stage III or IV) [21]. Our results demonstrated *MET* FISH-positivity has an impact on prognosis both in early TNM stage (stage I-II) and in advanced TNM stage (stage III-IVa). And there was no prognostic difference between stage I-II ESCC with *MET* FISH-positivity and stage III-IVa ESCC with *MET*-negativity. The findings indicate that *MET* gene alteration could be acquired during the early phase of ESCC development, and exaggerated the cancer progression [41].

## Conclusions

We investigated *MET* gene copy status using FISH, in a large series of ESCC. Our data show that increased *MET* gene copy number is an independent prognostic factor in surgically ESCC, and we firstly find that ESCC might have potentially been up-staged by increased *MET* gene copy number, which indicates increased *MET* gene copy number is a very promising parameter, in clinical therapy and follow-up plans.

## Abbreviations

AJCC: American Joint Committee on Cancer
DFS: Disease free survival
EAC: esophageal adenocarcinoma
EC: Esophageal cancer
EGFR: epidermal growth factor receptor
ESCC: esophageal squamous cell carcinoma
FFPE: formalin-fixed paraffin-embedded
FISH: fluorescence in situ hybridization
HGF: hepatocyte growth factor
MET: Mesenchymal epithelial transition
NSCLC: non-small cell lung cancer
OS: overall survival
RTK: tyrosine kinase activity
TKI: tyrosine kinase inhibitor
TMA: Tissue microarrays
